# A factorial experiment grounded in the multiphase optimization strategy to promote viral suppression among people who inject drugs on the Texas-Mexico border: a study protocol

**DOI:** 10.1186/s12889-023-15172-2

**Published:** 2023-02-10

**Authors:** John A. Sauceda, Julia Lechuga, Maria Elena Ramos, Jorge Puentes, Natasha Ludwig-Barron, Jorge Salazar, Katerina A. Christopoulos, Mallory O. Johnson, David Gomez, Rogelio Covarrubias, Joselyn Hernandez, David Montelongo, Alejandro Ortiz, Julian Rojas, Luisa Ramos, Itzia Avila, Marya V. Gwadz, Torsten B. Neilands

**Affiliations:** 1 Center for AIDS Prevention Studies, Department of Medicine, University of California, San Francisco, 550 16th Street, 94158 San Francisco, CA USA; 2 College of Health Sciences, Department of Public Health Sciences, University of Texas at El Paso, 1851 Wiggins Rd., 79968 El Paso, TX USA; 3 Program Compañeros, Cuidad Juárez, Avenue de la Raza 2661, Silvias, Chihuahua México; 4 Division of HIV, Infectious Diseases and Global Medicine, University of California, San Francisco, 996 Potrero Avenue, Building 80, 6th Floor, 94110 San Francisco, CA USA; 5 Centro de Integración Juvenil (CIJ), Cuidad Juárez, Blvd Ing Bernardo Norzagaray, 32130 Cazatecas, Chihuahua México; 6 Centro Ambulatorio para la prevención y Atención de SIDA y de las Enfermedades de Transmisión Sexual (CAPASITS), Cuidad Juárez, Avenue Paseo Triunfo de la Republica 3530, 32330 Partido Escobedo, Chihuahua México; 7 Silver School of Social Work, New York University, 1 Washington Square N, 10003 New York, NY USA

**Keywords:** Multiphase optimization strategy (MOST), HIV viral load suppression, HIV care continuum, Medication-assisted treatment (MAT), Behavioral activation therapy (BAT), Patient navigation, ART adherence, Life-steps, Factorial experiment, U.S.-Mexico Border

## Abstract

**Background:**

People who inject drugs living with HIV (PWIDLH) suffer the lowest rates of HIV viral suppression due to episodic injection drug use and poor mental health coupled with poor retention in HIV care. Approximately 44% of PWIDLH along the US-Mexico border are retained in care and only 24% are virally suppressed. This underserved region faces a potential explosion of transmission of HIV due to highly prevalent injection drug use. This protocol describes an optimization trial to promote sustained viral suppression among Spanish-speaking Latinx PWIDLH.

**Methods:**

The multiphase optimization strategy (MOST) is an engineering-inspired framework for designing and building optimized interventions and guides this intervention. The primary aim is to conduct a 2^4^ factorial experiment in which participants are randomized to one of 16 intervention conditions, with each condition comprising a different combination of four behavioral intervention components. The components are peer support for methadone uptake and persistence; behavioral activation therapy for depression; Life-Steps medication adherence counseling; and patient navigation for HIV care. Participants will complete a baseline survey, undergo intervention, and then return for 3-,6-,9-, and 12-month follow-up assessments. The primary outcome is sustained viral suppression, defined as viral loads of < 40 copies per mL at 6-,9-, and 12-month follow-up assessments. Results will yield effect sizes for each component and each additive and interactive combination of components. The research team and partners will make decisions about what constitutes the optimized multi-component intervention by judging the observed effect sizes, interactions, and statistical significance against real-world implementation constraints. The secondary aims are to test mediators and moderators of the component-to-outcome relationship at the 6-month follow-up assessment.

**Discussion:**

We are testing well-studied and available intervention components to support PWIDLH to reduce drug use and improve their mental health and engagement in HIV care. The intervention design will allow for a better understanding of how these components work in combination and can be optimized for the setting.

**Trial registration:**

This project was registered at clinicaltrials.gov (NCT05377463) on May 17th, 2022.

**Supplementary Information:**

The online version contains supplementary material available at 10.1186/s12889-023-15172-2.

## Background

The goal of HIV care and treatment is to achieve viral suppression. However, highly marginalized groups, such as people who inject drugs (PWID), face multiple and long-standing challenges to both achieving and sustaining viral suppression. In our Texas-Mexico Border setting, these challenges include episodic heavy use of opiates by injection [1–3], co-occurring untreated depression [4–6], non-adherence to oral antiretroviral therapy (ART) [7–9] and difficulty staying engaged in life-long HIV care [10–12]. For example, in one 13-year study of nearly 800 PWIDLH, 94% were initially linked to HIV care and 84% achieved viral suppression in the short term [10]. But over an 8-year period, only 33% stayed in HIV care continuously and only 10.2% remained virally suppressed [10]. Other longitudinal studies showed that approximately 50% of PWIDLH are not virally suppressed by one year after baseline study visits [2, 12]. Thus, PWIDLH are a high priority population for global HIV care and treatment research.

This manuscript describes a protocol for an intervention study in the Ciudad Juárez (CJ), Mexico - El Paso, Texas border region. These two cities are nestled immediately next to each other, cover a geographically vast area with a total population of 2.3 million people, and make up a primary corridor for illegal drugs, specifically heroin [13]. In CJ, the injection drug use rate is over four times the national average (22.3% vs. 3.4% in persons aged 12–65) [14]. CJ has an estimated HIV prevalence of 5.8% among PWID and 11.2% among women who inject drugs and engage in sex work [14–16]. CJ has among the lowest rates of viral suppression with an estimated point prevalence of 25% among PWIDLH [17]. While second in size to the San Diego-Tijuana Border, this region is largely underserved and has not received the same research attention it is owed.

Because sustaining viral suppression is difficult for PWIDLH, the behavioral intervention components we describe are targeting four risk factors: (1) heavy episodic injection drug use, (2) co-morbid depression, (3) a lack of knowledge and support to integrate ART into daily lives and routines, and (4) difficulty navigating health systems [1–12]. Our study aims to leverage four previously-studied intervention components by testing the components and then optimizing an efficient and effective multi-component intervention comprised of one of more of these four components.

The first component is medication for opioid use disorder (MOUD, also referred to as medication-assisted treatment) [18]. MOUD, such as methadone or buprenorphine, reduces physical dependency on opioids (i.e., withdrawal and cravings) and in one meta-analytic study was associated with a two-fold increase in ART adherence and 45% increase in the odds for viral suppression [19–23]. The second component is behavioral activation therapy (BAT), an efficacious and streamlined behavioral modification intervention for depression [24], as depression is highly comorbid with injection drug use [25]. Two RCTs showed BAT to be as effective as pharmacotherapy and cognitive-based therapies on reducing depressive symptoms [26, 27]. The third component is an adherence-promoting program called Life-Steps, which provides knowledge and skills to integrate HIV medications into daily routines [28]. Life-Steps and BAT programs have been integrated into depression intervention studies for PWIDLH and Latinx PLWH and demonstrated efficacy for improving HIV treatment adherence and depression [29, 30]. A 2017 meta-analysis showed that CBT-based and adherence-promoting interventions produced odds ratios of 1.45 and 1.28 for achieving viral suppression [31]. The fourth component is patient navigation, which has shown efficacy as an intervention to link PLWH with multiple comorbid conditions, including drug use, into HIV care. Independent studies and a review show patient navigation is strongly associated with viral suppression [32–34]. This protocol describes a study to optimize these four components for PWIDLH living on the CJ-El Paso Border, and then, based on results and the study’s “optimization objective” defined below, optimize an efficient multi-component intervention comprised of a subset of the components.

These components have been combined in various ways in several studies for PWIDLH [30, 35–38]. One recent example is HPTN 074 - a multi-site international study that used psychosocial counseling, substance use treatment, and referrals for HIV treatment for PWID [38]. While promising, there is a gap in knowledge when intervention components are tested as “packages” (i.e., all components combined and tested against a standard of care). Specifically, it is unknown which components in the package drove an effect, if one component diminished the effect of another component, or if fewer (e.g., two or three) components interact and produce an equal or greater effect than all components. Packaged interventions have also been labeled “black boxes” because their mechanisms of action are unclear even if they demonstrate efficacy [39]. Most concerning is that researchers could be wasting human and financial resources and cause undue participant burden if certain components add no value, diminish the impact of other components, or are iatrogenic. To overcome this scientific gap and leverage existing efficacy data, our experimental design follows the multiphase optimization strategy (MOST) - an engineering-inspired intervention framework [39].

MOST is a three-phase framework for building and optimizing interventions and approaches intervention science as engineers do product development. This includes phase 1, the preparation phase, which includes leveraging formative research and pilot data to design a conceptual model (akin to an engineering drawing) that theoretically defines all expected behavioral change mechanisms, and select the optimization objective. Then, in the optimization phase, an appropriate experimental design is chosen and applied for testing each component’s performance and each combination of components, and a decision making process is carried out to create or optimize the new multi-component intervention, taking into consideration implementation issues such as those related to a real-world context (e.g., staff availability and qualifications, justification of costs, participant burden) [40]. Last, depending on results, in the third phase the new optimized intervention can be tested in an RCT, or a return to an earlier phase in the framework may be warranted. One goal with MOST is to fully understand and test the inner workings of an intervention in a real-world setting.

### Objectives and aims

The objective of this protocol is to build an efficient, economical, and scalable optimized intervention to help sustain HIV viral suppression among a highly-underserved population of Spanish-speaking PWIDLH. We are leveraging existing resources and staff at partnering sites serving PWIDLH. As noted above, the components are: (1) peer support for MOUD uptake and persistence; (2) behavioral activation therapy for depression; (3) Life-Steps to support medication adherence [27], and (4) patient navigation for retention in HIV care.

**Fig. 1:**
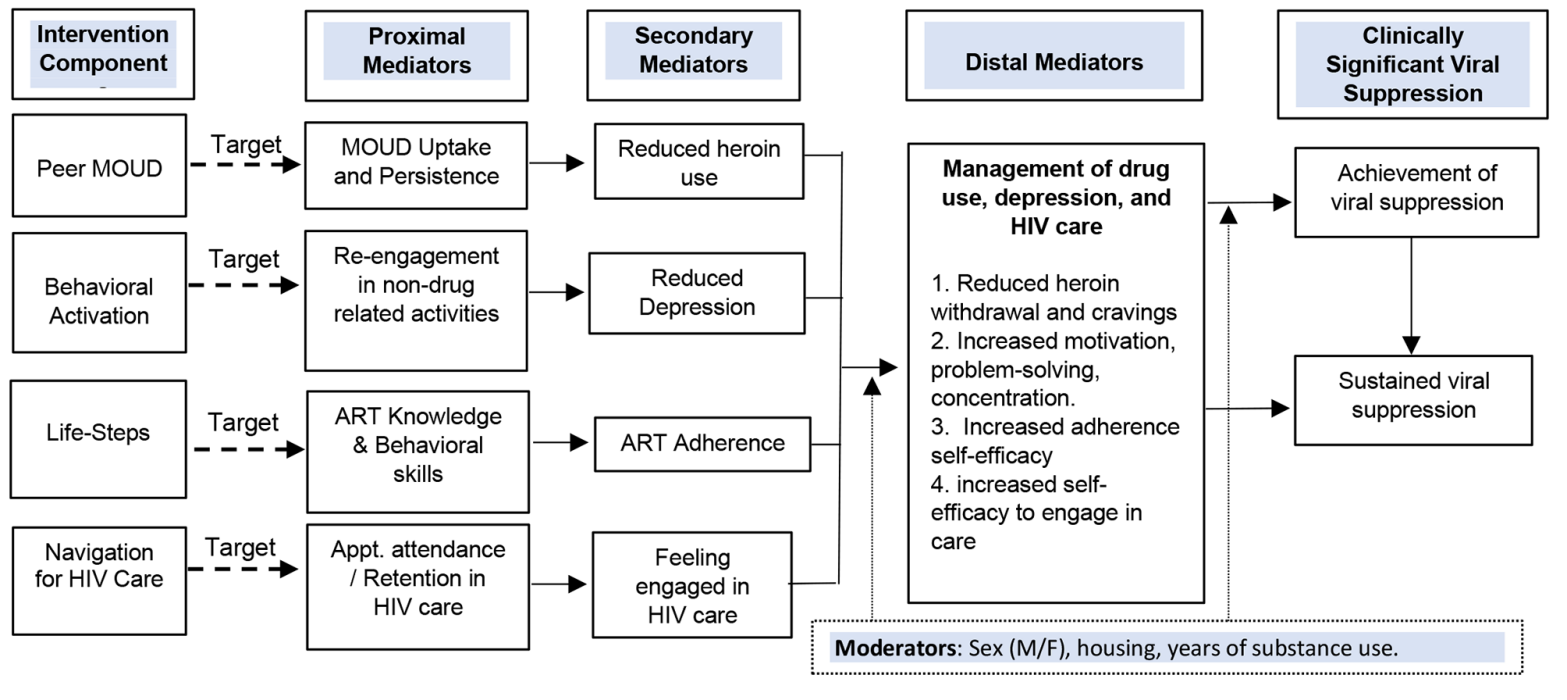
Conceptual Model

The primary aim is to conduct a factorial experiment (optimization trial) to estimate the main effects of each component and every combination of components (interactions) on the outcome of sustained viral suppression. The secondary aims are to test mediators that explain the relationship between each component and achievement of viral suppression, and to identify moderators of the relationship between each component and achievement of viral suppression (See Fig. 1).

## Methods/Design

### Ethics approval and consent to participate

The study and consent documents and processes have been approved by the Institutional Review Board at the University of Texas at El Paso. The study has also passed the review of ethical and data safety and monitoring plans from an independent Data and Safety Monitoring Board (DSMB) of Addiction Medicine at the University of California, Los Angeles (this site is independent of the authors/research team and their sites). The DSMB will meet annually to cover data monitoring activities (e.g., interim analyses, reporting adverse events, trial audit). The study does not use any animal or human data tissue and is registered at clinicaltrials.gov (NCT 05377463).

## Study setting

The Ciudad Juárez (CJ) - El Paso border region is a vibrant metropolitan area with high levels of in-and-out migration that also suffers from a fragile public health infrastructure and frequent acts of drug cartel violence [41]. Research shows that geographical borders are places where multiple factors interact to produce coexisting and mutually reinforcing risk environments that results in poor HIV treatment outcomes for PWIDLH [42, 43].

### Partnering Sites

The primary study site is *Programa Compañeros* (hereafter *Compañeros*), which is a community-based, harm reduction and social services organization that employs 30 staff members, including social workers, psychologists, physicians, nurses, peer navigators, and peer community outreach specialists. The director is a licensed social worker who has been involved in several studies with PWID.

The secondary sites are *CAPASITS* (pronounced – *ka-pa-seets*: Centro Ambulatorio para la Prevención y Atención en SIDA e Infecciones de Transmisión Sexual), which is an HIV and STI treatment clinic, and *CIJ* (pronounced *se-e-hota*: Centros de Integración Juvenil), which is a substance use treatment center. *CAPASITS* is the largest government run program providing HIV care and free HIV treatment in Mexico. CIJ is the largest program in Mexico offering substance use services, including out-patient, in-patient, and methadone treatment (buprenorphine is not available at CIJ).

## Overview & conceptual model

We have completed phase one (*preparation*) within the MOST framework. The *preparation* phase activities included leveraging preliminary and formative intervention and qualitative data, literature reviews, expert consultations, and existing programs and staff skills at our primary site (e.g., patient navigation services, staff psychologists) [44]. The selection of components was based on services currently available but not fully optimized in the sites and the area (i.e., access to MOUD and free ART, staff psychologists who deliver therapy). We created a conceptual model specifying the hypothesized causal processes and moderators for each intervention component, which allows for falsifying or confirming the full causal processes in the intervention. We will then diagnose weak performing components and identify areas for refinements by testing mediators and moderators of each component pathway.

For the *optimization* phase (phase two), we chose a 2^4^ factorial experiment and defined our optimization objective, which is the required operational definition of what we mean by the idea of optimization. Our optimization objective [40, 45] is the best combination of components for viral suppression for PWIDLH under three real-world constraints. The three constraints identified in MOST and in this protocol are evaluations about efficiency (did the components perform well with existing staff at the organization?), economy (did the observed effect sizes justify the costs?), and scalability (did it perform exactly as it would at scale?) [40]. The present protocol comprises the *optimization phase*. After the factorial experiment is completed, components will be evaluated not only by the observed effect sizes and statistical significance, but against any constraints imposed by the implementing setting. The goal is to arrive at an optimized intervention (V.1.0) that is not a prototype, but more likely to be an efficient and effective intervention ready to be scaled.

## Trial Design

**Fig. 2:**
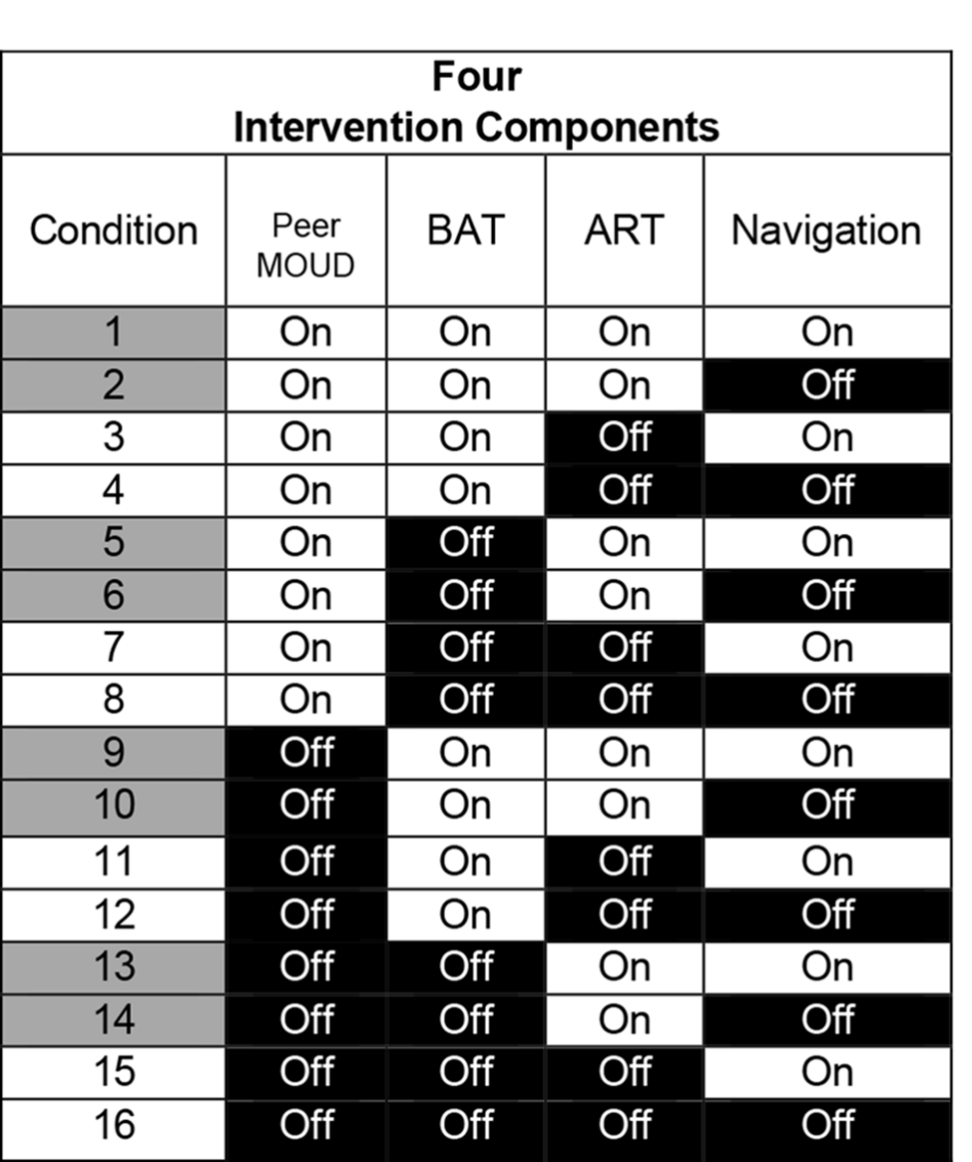
2^4^ Factorial Design Figure 2 shows the efficiency of the factorial design. Aggregated cell proportions of HIV viral suppression of different combinations are used to estimate effects. On = delivered, Off = not delivered. Ex. 1. Gray colored vs. white colored conditions on left side show how we compare the effect Life-Steps for medication adherence (ART).

The trial is a 2^4^ factorial design (see Fig. 2). The four components are set to two levels (1 = on or 0 = off). Thus, a 2^4^ design equates to 16 unique conditions. The 16 conditions represent every combination of the four components. The number of conditions allows for main and interactive effects on the outcome to be estimated [46]. Interactive effects can be additive, synergistic, or antagonist relationships among the components [47]. These effects inform optimization decisions and show the combinations that were beneficial, harmful, or inefficacious.

## Randomization

Fully balanced factorial designs, in which there are equal *N*s per cell in each condition (see Fig. 1), maximize statistical power. To achieve balance, participants will be placed in a permuted block and assigned to each of the 16 conditions at random after baseline assessment. Repeated blocks will be created so no one condition (1–16) is filled with more participants prior to the others, leading to potential imbalances [48]. Stratification will be based on the prognostic characteristics of sex in this population (85% men, 15% women) to ensure balance across conditions and aid in generalizability. Participants assigned to condition 16, which has no components but is not considered a control group, will be given the standard of care in CJ (i.e., support groups) and an enhanced referral program that all participants can receive.

## Description of four intervention components

### Component 1 – peer-support for MOUD uptake and persistence

Two certified peer recovery coaches staffed by *Compañeros* will deliver two informational sessions, a tailored recovery plan, and ongoing support from a peer for the first six months [49, 50]. The intervention protocol is based on the SAMHSA peer recovery services manual [51]. Peers will provide information about methadone and the brain, including mechanisms of action, methadone myths and safe use, and patient self-advocacy [23, 51]. Peers will engage participants in a conversation about the benefits of methadone, strategies to adhere and manage side effects, and strategies for reducing stigma. Peers will assist in enrollment by scheduling an appointment and accompanying the participant in collecting and submitting required documentation to CIJ.

### Component 2 – behavioral activation therapy for Depression

Two staff psychologists at *Compañeros* will deliver BAT as a modified 8-session manualized program for depression [52]. The BAT program will identify short and long-term goals and values and the activities unrelated to substance use that were previously enjoyed or liked to increase a feeling of well-being. Weekly sessions include psychoeducation on depression, the BAT rationale, the activities and monitoring of activities, building social contracts to engage networks, and discussions of barriers that may interfere with activities.

### Component 3 – life-steps for medication adherence

A research assistant for special projects and a coordinator of initiatives for PWID at *Compañeros* will deliver Life-Steps as a single session with one follow-up phone call or visit [28]. The manualized content is based on cognitive-behavioral techniques and consists of teaching the importance of HIV treatment and the strategies and skills needed to take HIV treatment as prescribed. The session consists of strategies for problem solving barriers, building communication skills to improve patient-provider interactions, coping strategies for side-effects, reminder cues and managing lapses.

### Component 4 - patient navigation for linkage and retention in HIV care

Two trained navigators staffed at *Compañeros* will provide information about the process of linking to HIV care, ART, and provide instrumental support to access care as needed during the first six months [33, 53]. The navigator will assist with obtaining government-sponsored insurance if the patient does not have employer-sponsored insurance, including processing the documents, monetary resources, transportation, and clerical support to obtain required documents, such as state ID and birth certificates. Navigators meet with participants to explain what can be expected during their clinic appointment and, in lay terms, medical terminology likely to be discussed by providers to facilitate patient-provider interaction. Other instrumental support provided will be scheduling an appointment at *CAPASITS*, providing clean clothing, a place to shower, and transportation to attend medical appointments.

#### Outcomes

The primary outcome is *sustained* viral suppression [54], which is a measure of viral load constancy. Sustained viral suppression is operationally defined as confirmed viral load laboratory tests of < 40 copies per mL (based on laboratory sites threshold) at 6-,9-, and 12-month follow-up assessments. Missing viral load data and deaths are treated as non-suppression and failure (details on plans for viral load testing are described below) [37]. Participants who are virally suppressed on all follow-up assessments are coded as 1 for sustained viral suppression. Participants who have missing data, died (all-cause mortality), or are not virally suppressed on one or more follow-up assessment(s) are coded as 0 for not having sustained viral suppression. This handling of missing outcome data allows for calculating the proportional differences between those who did and did not sustain viral suppression using an intent-to-treat approach [55].

The secondary outcome for our secondary analyses is *achievement* of viral suppression, which is operationally defined as confirmed VL test < 40 copies per mL at the first 6-month follow-up assessment [56]. Survey data on all mediating variables (X to M to Y) will be collected during a second survey assessment 90 days (M) after the baseline assessment (X) and after the delivery of all components, but before the 6-month follow-up assessment (Y). All missing survey data will be assessed for missingness assumptions and multiple imputation will be used to handle missingness as appropriate [57].

## Participant recruitment

### Eligibility criteria

Inclusion criteria were modeled after two large intervention studies for PWIDLH [35, 37]. Inclusion criteria are: (1) be 18 years of age or older; (2) living with HIV, (3) injected drugs in the last 30 days; (4) screen positive for depression on the patient health questionnaire [58]; (5) be willing to meet with peer educator about MOUD; (6) sign a medical data release form; (7) agree to provide and update locator information; (8) agree to return for follow-up study visits; (9) be able to communicate in Spanish; (10) be eligible to receive free federal services for HIV care in Mexico; 11) be able to provide informed consent; 12) and meet one of the following HIV treatment-related criteria: (a) not in possession of ART or not taking prescribed ART; or (b) sub-optimal ART adherence defined as at least one 4-day treatment interruption in the past 90-days [59]; or (c) sub-optimal retention in HIV care - never engaged or disengaged from HIV care, defined as two or more missed clinic appointments in the previous nine months; or (d) no viral load test in the past six months or self-reports a detectable viral load within the past six months. All medical record data is verifiable through *CAPASITS* or will be confirmed through a private laboratory.

### Participant recruitment and screening

We are using three recruitment strategies. First is in-person recruitment at *Compañeros*. Approximately 5,000 PWID visit *Compañeros* each year, which can exceed 100 PWID each day. *Compañeros* is located in a discreet location in central CJ. Upon entering the primary site, individuals will be screened during their check-ins with staff, which is a protocol in place for all services provided. If screened positive, we will direct interested individuals to the full-time study coordinator who will complete the eligibility assessment in a private study office.

Second is recruitment through direct outreach. There are large numbers of injection drug use sites across 200 neighborhoods in CJ. Every week, *Compañeros* staff visit approximately 25 sites in a mobile clinic van to deliver harm reduction supplies. *Compañeros* has engendered a deep trust with PWID, allowing staff members to administer brief surveys on site. Staff members will screen for self-reported current injection drug use, depression, and HIV status. A positive screen will trigger a follow-up protocol, which includes transportation to *Compañeros* for services and an eligibility assessment.

The final recruitment strategy is Respondent Driven Sampling (RDS) [60, 61]. Staff will recruit potential seeds during normal outreach activities (i.e., delivery of prevention care kits, testing, etc.). Participants who are eligible to participate and volunteer as seeds will be provided with three trackable coupons. Seeds will distribute coupons to refer three peers who may meet eligibility criteria. Referred participants will be screened for eligibility at *Compañeros*. After participants recruited by the seeds are enrolled, they may also recruit from their own networks. Participants will receive $10 for each participant they successfully recruit. Extra coupons will be provided to participants who turn out to be successful recruiters.

#### Screening and enrollment on-site at compañeros

Full eligibility screening will take place in our private room at *Compañeros*. After eligibility criteria is ascertained, participants will be provided with the consent form and complete the informed consent process. As part of this process, participants will be asked to consent to verification of HIV status and suboptimal HIV care from abstracted medical records. If no records exist, participants will be asked to consent to an HIV test and confirmatory viral load test through our partnership with a private laboratory next to *Compañeros*. Costs for the private laboratory viral load testing are provided by the study. Participants will then be scheduled for a second visit to conduct the baseline assessment and randomization procedures, contingent upon confirmation of eligibility criteria.

### Fidelity monitoring for interventions and quality assurance of study data

We adapted a framework for measuring multiple dimensions of fidelity [62]. The dimensions include: (1) measuring adherence to the intervention protocol, (2) measuring quality of intervention delivery, (3) measuring whether participants responded to the intervention as intended, and (4) whether the different intervention groups were distinct and did not show contamination across conditions. For each component, we will: (1) assess component adherence via audio recordings and reviews of checklists and observations; (2) define performance criteria precisely and train interventionists to follow performance criteria; (3) provide booster training sessions biannually to ensure skills do not decay over time; and (4) address setbacks in implementation by training additional staff members as alternates in case of illnesses or other unanticipated personal events. Fidelity is assessed during a scheduled monthly group and individual supervision sessions. This framework aims to ensure that *theoretical causes of change* (our conceptual model) are carried out as *actual causes of change* (intervention executed as planned) [62].

#### Contamination across conditions

All interventionists will use checklists to document intervention delivery and type session notes for easy routine access by supervisors during their evaluation. Further, all interventionists are only assigned to deliver one intervention component (rather than multiple components). We will use a separate electronic and paper system for enrolling participants into the trial and assigning them to their condition to avoid mis-assignment to conditions due to human error, and we will ask participants not to discuss the details of their intervention sessions and processes with other individuals until the study is over.

For quality assurance of data, study staff will be responsible for collecting and tracking assessments and conducting an initial verification of data quality. The study coordinator and data manager will enter, review and re-verify the survey data. After this verification process, the two principal investigators will review all data weekly by creating statistical software code to generate frequency tables for all variables, and measures of central tendency and variability for continuous variables to characterize the sample and identify outliers. These quality control steps are necessary to evaluate the quality of the survey data being collected and for missing data. All program code will be documented extensively to enable future code review, transparency, and reproducibility of results.

For quality assurance of clinical data, we have outlined in a scope of work with partners at the HIV clinic and substance use treatment center that they are to report data within a two-week window of time (before or after), which is based on each follow-up assessment. These data include viral load test results, HIV care appointment attendance records, ART prescription data, and any records of death, uptake and persistence with MAT, and urine analyses. The director of the primary site, *Compañeros*, will be responsible for managing local quality assurance of clinical data on a regular basis and report any problems or delays to principal investigators and our Data and Safety Monitoring Board.

## Data collection

### Primary and secondary outcomes

Viral load data will be collected and verified through *CAPASITS* - the primary HIV clinic. In a situation where viral load testing cannot be done or collected, the study has budgeted for transportation and testing from a local private diagnostic laboratory. A database maintained in Research Electronic Data Capture (REDCap), a password protected and data encrypted computerized interface, will be used to enter data from paper medical records and reports into an electronic form.

### Surveys

The baseline self-report survey consists of all key measures in the conceptual model (see Fig. 1). All surveys will be administered by study staff. In addition to demographic information, the survey will collect data on mediators (drug use frequency and quantity of injection drug use, opioid withdrawal and craving symptoms, depression symptom ratings, ART adherence and adherence self-efficacy, ART knowledge, and HIV care barriers and perceived engagement with HIV care). Data on MOUD uptake and persistence, as well as clinic appointment attendance will be abstracted from medical charts at *CAPASITS* and *CIJ*. All surveys will be completed using paper-and-pencil and later entered into the REDCap datafile. Each row of data will be read and verified by two staff members.

## Follow-up plan and tracking

Our study will require follow-up assessments over 12 months. We will conduct assessments at baseline, 3-month, 6-month, 9-month, and 12-month visits. We will remunerate participants for their time with a graded system of payments for follow-up assessments on-site at *Compañeros*. Participants are given $20 for the baseline survey, $25 at the 3-month follow-up assessment, $35 at 6-month follow-up assessment, and $45 at the 9- and 12-month follow-up assessments, plus a bonus of $25 for completing all assessments (USD amounts will be converted to Mexican pesos).

Tracking is accomplished by using a detailed locator information form and by maintaining frequent contact with study participants. Locator information will be updated each time there is contact with participants. We will keep and maintain a computerized tracking system at every participant contact. Some participants will have unstable housing and will require active outreach follow-up activities by *Compañeros’* staff in community venues or drug use sites identified from locator forms.

### Harms

Potential harms are breaches of confidentiality, embarrassment, discomfort, or distressed emotional reactions to the study assessment measures and/or intervention topics, particularly those related to depression and drug use; increased anxiety concerning HIV; increased anxiety regarding drug use problems and need for treatment; and disruption in risk-producing social relationships as a result of participants’ behavior change efforts. There is a risk that the interventions may not work. The risks are detailed and monitored by the Data and Safety Monitoring Board, which approved the study protocol in June of 2022.

### Data analytic plan

The primary aim is to estimate main and interaction effects for sustained viral suppression. For analyses with clustering (within-subject correlations) and based on a revised analysis plan from the Data and Safety Monitoring Board, we modified our plan to fit a three-level generalized linear mixed model (GLMM) [63]. GLMM will be used to account for the nestedness of person within repeated assessments and person within respondent-driven sampling seeds [63], with the probability of viral suppression from each time point averaged across the different treatment conditions and including the baseline assessment as part of the outcome. Baseline viral load will also be collected and analyzed and alpha (α) will be set at 0.05 for estimating all main effects. Lastly, as detailed by Kahan and Morris (2011), stratifications during the randomization process must be accounted for in the primary analysis as it may lead to correlations among the participants in each cell in each of the 16 conditions [64]. Therefore, all analyses will be adjusted by sex to produce unbiased estimates.

For interactions, they will be estimated on the primary outcome. Guidance from VanderWeele & Knol (2014) [65] and Knol (2007) [47] detail the nuances of both additive and multiplicative (synergistic/antagonistic) interactions that are necessary to understand how best to optimize the intervention. In line with guidance from the developer of the MOST framework, alpha will be set to 0.10 for all interaction effects, which is justified under the decision-priority perspective below [66].

### Decision-priority perspective - defining the final optimized intervention

In MOST, the goal is to build an optimized intervention with the data the investigative team has on hand. One approach to building an intervention is the use of the *conclusion-priority perspective*, where optimization is based solely on the statistical significance at *p* < .05 of each component (rejected the null hypothesis) [66]. However, if a component or an interaction effect failed to reject the null hypothesis, it is not known whether that component or that interaction genuinely has no effect on viral suppression or if an incorrect rejection was made (Type II error). This conclusion may erroneously suggest that one or more components should not be included in the optimized intervention and voids the purpose of the study. In a *decision-priority perspective*, the components and interactions that evidence a statistically significant effect may or may not be included in the optimized intervention as their inclusion depends on how they performed under real-world constraints (i.e., efficiency, economy, and scalability) [66]. For example, if the difference in the odds for viral suppression between 3 and 4 components is 5%, the investigative team and our partners could decide that the difference is not worth the burden, time, or cost to implement the fourth component. Alternatively, three components could synergistically interact and produce an effect equivalent to having all four components delivered. Thus, to ensure we have full information for optimization, we can reduce the risk of making an incorrect rejection (Type II error) by setting alpha to 0.10 for detecting interactions. All interactions will be graphed to visualize their effects. Final decisions about what constitutes the best expected outcome and constraints are judged by dissemination meetings by the investigative team and our partners [40].

#### Analysis plan for secondary aims

The secondary aims are to conduct mediation and moderation analyses on viral suppression at 6-month follow-up assessment. For our mediation effects, we will generate estimates of the mediated effects and bootstrapped samples yielding 1000 randomly generated (*k* = 1000) to approximate the empirically derived sampling distribution that is then used to create a 95% confidence interval around the mediation effect [67]. This, along with heteroscedasticity-consistent standard errors and a bias correction for the confidence intervals, is the primary mode to determine the presence of mediation [67].

For moderation effects, principles of modern moderation analyses for categorical and continuous variables will be followed [68, 69]. For rigor and transparency, we will first characterize a moderating effect using the percentile approach that estimates the effect sizes across different values of the moderator [70]. For all continuous measures of moderating variables, we will probe for interactions using the Johnson-Neyman Technique [69]. This technique derives statistically significant estimates along the continuum of values of the moderating variable. Through this approach, we will estimate regions of statistical significance that provide an understanding of the moderator values (e.g., high, medium, low) that change the component-outcome relationship.

## Power analysis

Power analyses were generated using the multilevel logistic regression module for proportions in a 2-level hierarchical design in NCSS PASS 19 [71]. We employed the hierarchical design module to account for the clustering present as a result of the use of RDS to recruit 1/3 of participants. Given the efficiency of factorial designs, we used the smallest effect size from the four components available in the literature in the power analysis [72]. We varied baseline rates of viral suppression from 30 to 50% based on regional data estimates available [17]. We set α = 0.05 and power = 0.80, and varied the intracluster correlation due to RDS from 0.03 to 0.08 based on estimates available in the literature [73], which yielded a minimum sample size of 320 to detect an estimated odds ratio of 2.0 corresponding to the smallest detectable difference in the primary outcome of sustained viral suppression at the 12-month follow-up. Benchmarks for standardized effect sizes are 0.20, 0.50, and 0.80 for small, medium, and large effect sizes, respectively [74]. The estimated odds ratio represents a minimum proportional difference of 16% and a standardized effect size of 0.31, which is commensurate with a small to medium effect size [74]. Estimating that approximately 20% of participants will be lost at follow-up, a minimum sample size of 384 will be required.

For secondary aims, with a sample of 384 participants, we are fully powered for our mediation hypotheses. Using a published power table for mediation [75], assuming regression paths (α and β) halfway between small and medium size (0.26, or range of variance accounted from 2 to 13%), which is conservative, and if individual effects (a path, b path, c path) were adequately powered (> 0.80), a sample size of 148 participants is needed.

## Discussion

Our study has the potential to significantly advance intervention science for PWIDLH and HIV care and treatment research. We are using four components that have been extensively studied by our team and others; however, we will test each component separately and in every combination to answer a novel research question. Furthermore, we are harnessing local infrastructure and on-going community programs to enhance the sustainability of the optimized intervention.

Operationally, there may be difficulties in screening large numbers of PWID and with study retention. Thus, we are using procedures that have been effective, including an incentive payment system and covering transportation costs. We will also obtain detailed locator information, deliver reminder phone calls, and conduct active outreach in community venues or drug use sites recorded within locator forms. Other operational challenges include participants not being interested in methadone or having minimal readiness for methadone, missing viral load data and varying completion of intervention components. To maximize interest and completion of intervention activities, all participants will continue to have access to services at *Compañeros* as part of a standard harm reduction package. *Compañeros* is a trusted key source of support for PWID and by leveraging staff and their expertise, we hope to maintain high interest in our study.

## Conclusion and impact

Sustaining viral suppression remains one of the biggest challenges in HIV research for PWIDLH. We propose to fill an important intervention research gap by carefully building an optimized multi-component intervention to address the multiple barriers faced by PWIDLH to promote sustained viral suppression in a high-risk setting. Our approach has the potential to advance the science by paving the way for other researchers to build directly from our conceptual model and evidence-base in a way that is systematic, transparent and forward thinking.

## Electronic supplementary material

Below is the link to the electronic supplementary material.

Supplementary Material 1

## Data Availability

Not applicable.

## Abbreviations

ART: Antiretroviral Therapy
BAT: Behavioral Activation Therapy
MOUD: Medication for Opioid Use Disorder
MAT: Medication-Assisted Treatment
MOST: Multiphase Optimization Strategy
REDCap: Research Electronic Data Capture
RDS: Respondent-Driven Sampling.
